# Clinical Evaluation of an Arterial-Spin-Labeling Product Sequence in Steno-Occlusive Disease of the Brain

**DOI:** 10.1371/journal.pone.0087143

**Published:** 2014-02-06

**Authors:** Matthias A. Mutke, Vince I. Madai, Federico C. von Samson-Himmelstjerna, Olivier Zaro Weber, Gajanan S. Revankar, Steve Z. Martin, Katharina L. Stengl, Miriam Bauer, Stefan Hetzer, Matthias Günther, Jan Sobesky

**Affiliations:** 1 Center for Stroke Research, Charité Universitätsmedizin, Berlin, Germany; 2 Fraunhofer/MEVIS, Bremen, Germany; 3 Berlin Center for Advanced Neuroimaging, Berlin, Germany; 4 Max-Planck-Institute for Neurological Research, Cologne, Germany; 5 Department of Neurology, Charité Universitätsmedizin, Berlin, Germany; University Medical Center (UMC) Utrecht, Netherlands

## Abstract

**Introduction:**

In brain perfusion imaging, arterial spin labeling (ASL) is a noninvasive alternative to dynamic susceptibility contrast-magnetic resonance imaging (DSC-MRI). For clinical imaging, only product sequences can be used. We therefore analyzed the performance of a product sequence (PICORE-PASL) included in an MRI software-package compared with DSC-MRI in patients with steno-occlusion of the MCA or ICA >70%.

**Methods:**

Images were acquired on a 3T MRI system and qualitatively analyzed by 3 raters. For a quantitative analysis, cortical ROIs were placed in co-registered ASL and DSC images. Pooled data for ASL-cerebral blood flow (CBF) and DSC-CBF were analyzed by Spearman’s correlation and the Bland-Altman (BA)-plot.

**Results:**

In 28 patients, 11 ASL studies were uninterpretable due to patient motion. Of the remaining patients, 71% showed signs of delayed tracer arrival. A weak correlation for DSC-relCBF vs ASL-relCBF (r = 0.24) and a large spread of values in the BA-plot owing to unreliable CBF-measurement was found.

**Conclusion:**

The PICORE ASL product sequence is sensitive for estimation of delayed tracer arrival, but cannot be recommended to measure CBF in steno-occlusive disease. ASL-sequences that are less sensitive to patient motion and correcting for delayed blood flow should be available in the clinical setting.

## Introduction

Cerebral blood flow (CBF) is an important measure of brain perfusion in patients with steno-occlusive disease [1]. Using magnetic resonance imaging (MRI), dynamic susceptibility-weighted contrast-enhanced (DSC) imaging is the clinical standard of CBF measurement. It is fast and offers a reliable estimate of CBF [2]. However, the main drawback of DSC imaging is the need of a gadolinium-based contrast agent. Administration of gadolinium is invasive, may cause anaphylaxis and patients with renal insufficiency must be excluded owing to possible nephrogenic systemic sclerosis [3]. Additionally, repeated measurements are difficult to perform owing to slow clearance of gadolinium based contrast agents [4]. Arterial spin labeling (ASL) measures CBF without the need of an exogenous contrast agent [5]. Here, blood is magnetically labeled by the MR scanner and the labeled blood water protons serve as an endogenous contrast agent to calculate CBF. ASL therefore permits multiple noninvasive CBF measurement. Various ASL techniques have been successfully validated to measure CBF in neuropathologies, e.g. in brain tumors [6], epilepsy [7] and stenosis of cerebral arteries and acute stroke [8]. Most of these sequences, however, were research sequences and are not available for clinical imaging. For clinical diagnosis, in contrast, available product sequences have to be used and their validation is urgently needed [9], [10]. We therefore studied the performance of a clinically available commercial ASL-sequence to measure CBF in comparison with DSC-MRI in patients with steno-occlusive disease of the brain.

## Materials and Methods

### Ethics Statement

All patients gave informed written consent prior to the study. The study was conducted according to the principles expressed in the Declaration of Helsinki and was approved by the authorized institutional review board (IRB) of the Charité - Universitätsmedizin Berlin.

### Study Design

We performed an observational prospective imaging study (Perfusion imaging by arterial spin labeling for clinical use in stroke - PEGASUS, WHO international Clinical trials registry No. DRKS00003198). Patients with steno-occlusive disease were recruited at the Department of Neurology of the Charité - Universitätsmedizin Berlin or presenting at our out-patient services between September 2011 and November 2012. Inclusion criteria were: a) unilateral stenosis >70% of one internal carotid artery (ICA) or middle cerebral artery (MCA) according to the ECST (European Carotid Surgery Trial) criteria, b) age 18–80 and c) clinically and hemodynamically stable status. Grading of stenosis was confirmed prior to MRI by Duplex-Sonography and/or CT-angiography.

Exclusion criteria were a) contralateral stenosis >50% of the CCA (common carotid artery), ICA or MCA b) magnetic implants, c) claustrophobia, d) aphasia or reduced level of consciousness, e) severe allergic reactions in the previous medical history, f) allergic reactions against Gadolinium-based contrast agents in the past, g) renal insufficiency (defined by a GFR ≤30 ml/min/1,73 m^2^), h) pregnancy and i) unstable clinical status. For each patient National Institute of Health Stroke Scale (NIHSS), modified Rankin Scale (mRS) and relevant clinical data was assessed before imaging.

### Magnetic Resonance Imaging Hardware

MR-imaging was performed on a 3 T whole-body system (Magnetom Trio, Siemens Healthcare, Erlangen, Germany) using a 12-channel receive RF coil (Siemens Healthcare, Erlangen, Germany) tailored for head imaging.

### Magnetic Resonance Imaging Sequences

We used a commercially available pulsed arterial spin labeling (PASL) PICORE Q2TIPS product sequence [11], [12]: 50 pairs of label/control ASL images were acquired in axial direction at a single inversion time of 1800 ms (EPI-readout, TR = 2600 ms, TE = 13 ms, TI1 = 700 ms, label thickness = 100 mm, PICORE Q2T perfusion mode, voxel size: 3×3×5 mm^3^, no crusher gradients, enabled prospective motion correction [3D-PACE]).

The DSC-MRI protocol consisted of 80 series of whole-brain images using a single-shot FID-EPI sequence (TR = 1390 ms, TE = 29 ms, voxel size: 1.8×1.8×5 mm^3^) after injection of 5 ml Gadovist® (Gadobutrol, 1 M, Bayer Schering Pharma AG, Berlin) followed by 25 ml saline flush by a power injector (Spectris, Medrad Inc., Warrendale PA, USA) at a rate of 5 ml/s. Acquisition time for ASL was 4 min:18 s, for DSC-MRI 1 min:54 s. DSC-MRI was performed immediately after ASL.

### Data Postprocessing

ASL images were acquired directly from the console and were used without further post-processing. DSC-MRI images were postprocessed offline with PMA (Perfusion Mismatch Analyzer, ASIST Japan, Iwate, Japan). Two maps were generated from the raw data: 1) DSC-time-to-peak (TTP)-maps -indicating regional delayed blood flow- and 2) DSC-CBF-maps – indicating regional blood flow - with automatic placement of the arterial input function using sSVD (standard singular value decomposition) deconvolution.

### Qualitative Analysis

Maps of ASL-CBF, DSC-CBF and DSC-TTP were visually assessed by three readers blinded to clinical data (JS, 10 years experience in stroke perfusion imaging; VM, 3 years; MM, 2 years; all 3 readers have special expertise in ASL imaging).

A majority rating was reached if at least two raters applied the same rating. In cases where all three raters disagreed, all three obtained a consensus.

**Figure 1:**
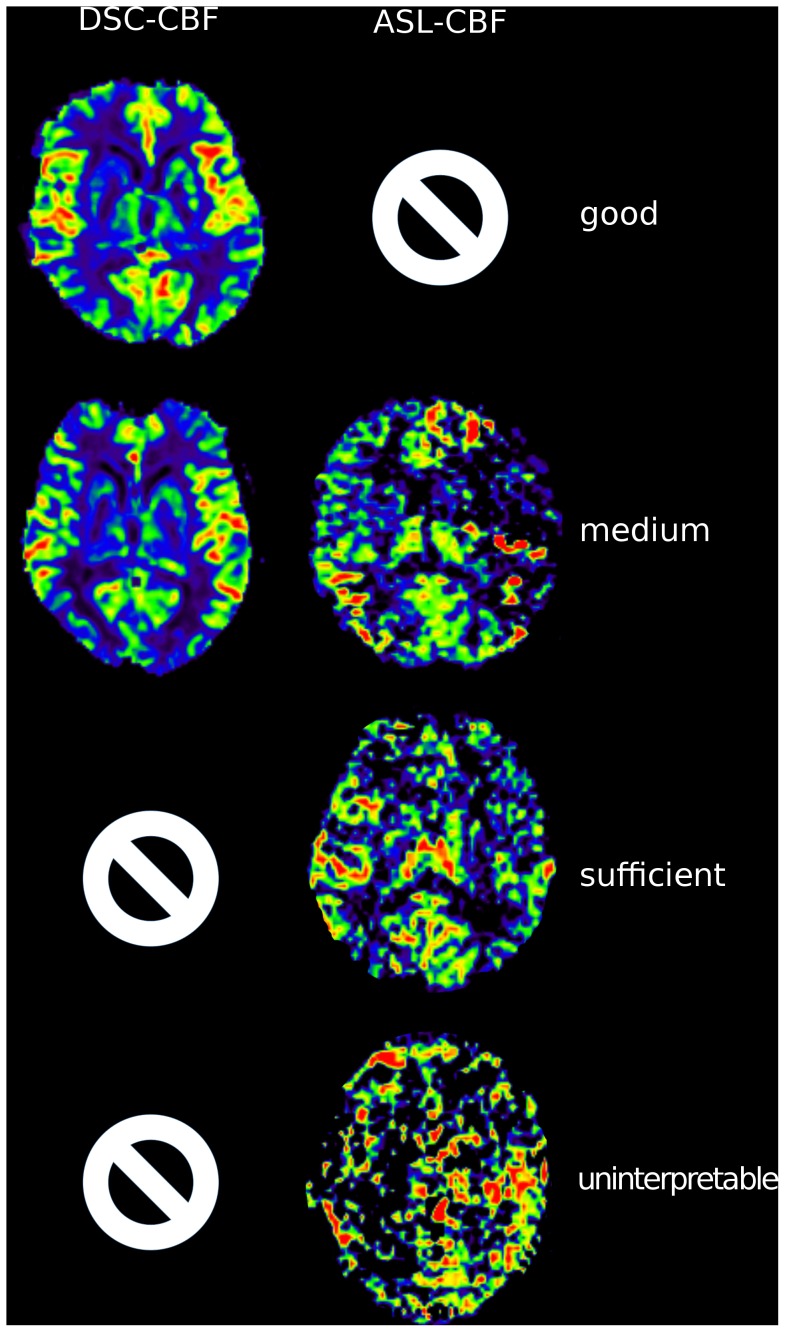
Rating of quality. The figure demonstrates the different image qualities. Rows show examples for good, medium, sufficient and uninterpretable quality. The first column demonstrates DSC-CBF images. All DSC-CBF maps were of either “good” or “medium” quality. The second column shows ASL-CBF images. No ASL-CBF map was rated “good”.

The rating was performed following a predefined standardized algorithm:

Imaging quality was rated as “good”, “medium”, “sufficient” or “uninterpretable” (Figure 1). Raters were asked to rate hyperintensities, hypointensities or normal intensity for each hemisphere on maps of ASL-CBF, DSC-CBF and DSC-TTP. On ASL-CBF images, raters were asked to note the appearance of artifacts owing to increased blood transit times, the “arterial transit delay artifact” (ATDA): It is defined by two signs: First, the occurrence of hyperintense lines or dots. Second, larger areas of severe hypoperfusion adjacent to the hyperintense areas [13]. (Both signs are are addressed in detail in the discussion).

### Quantitative Analysis

For the quantitative analysis, ASL-images rated as “uninterpretable” were excluded. Coregistration and region of interest (ROI) placement was performed with VINCI 3.93 (Max-Planck Institute for Neurological Research, Cologne, Germany) [14].

**Figure 2:**
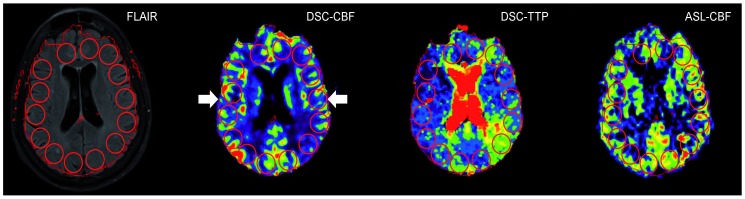
Quantitative analysis of DSC-CBF, DSC-TTP and ASL-relCBF. 20(ROIs) were placed on the FLAIR image and copied onto coregistered DSC-CBF, DSC-TTP and ASL images. For relative values, the ratio of each ipsilateral ROI to the arithmetic mean of all contralateral ROIs was calculated. In case of DSC-TTP the values were subtracted to obtain relative TTP-delay. DSC-perfusion maps were automatically masked by the used software. Therefore, the contour of DSC maps was used to define the cortical rim (contour, arrows).

Maps of ASL-CBF, DSC-CBF and DSC-TTP were coregistered with FLAIR as the reference image. Cortical ROIs (diameter: 20 mm) were placed on 7 consecutive axial slices located at the height of the lateral ventricles. Slices above the lateral ventricles and slices including the cerebellum were not included. ROIs were placed on FLAIR images and copied on all perfusion maps (Figure 2). ROIs were labeled according to the side of steno-occlusion (ipsi- or contralateral). Relative CBF values (relCBF) were calculated using the mean value of each ROI: (ipsilateral ROI value/mean of all contralateral ROIs on the same slice) [%]. The relative time to peak (relTTP) for ipsilateral ROIs was obtained by subtraction from the mean TTP of all contralateral ROIs. Negative voxel values were not included in the analysis.

In a following ROI-analysis, TTP-ROIs were divided in two groups: First, TTP-ROIs showing an ATDA in the corresponding ASL-ROI and second TTP-ROIs without ATDA in the corresponding ASL-ROI. For comparison, the relative TTP delay was calculated as described above.

In a further volumetric analysis, we segmented grey matter on MPRAGE (T1) maps using Mevislab (MeVis Medical Solutions AG, Bremen, Germany). The grey matter map was divided into three flow territories: ACA (anterior cerebral artery), MCA (middle cerebral artery), PCA (posterior cerebral artery) based on a brain perfusion atlas [15]. The grey matter maps were applied to co-registered ASL-CBF and DSC-CBF maps. The average value for a specific flow territory volume was normalized to the mean perfusion of the whole contralateral grey matter perfusion.

### Statistical Analysis

Study data are given in median and interquartile range owing to skewed distribution of some variables if not indicated otherwise. In the ROI-analysis, values of ASL-relCBF were plotted against relTTP and DSC-relCBF and correlated using Spearman’s rho. The Bland-Altman (BA) plot was used to compare values of ASL-relCBF vs. DSC-relCBF. Logarithmic BA plots were used, when measurement errors seemed to be proportional to the mean.

For the comparison of the relative TTP-delay of TTP-ROIs with ATDA vs. without ATDA on corresponding ROIs in ASL we used the Man-Whitney U test, since the distribution was skewed.

In the analysis of the grey matter perfusion, values of ASL-CBF and DSC-CBF were compared using Spearman’s rho.

All statistical methods described were applied to pooled data from all patients. Statistical tests were performed with SigmaPlot 11 (Systat, San José, California, U.S.A.).

## Results

28 patients met the inclusion criteria, all of them completed the imaging protocol.

Median age of all patients was 58 years, 20 patients had previous stroke or TIA. Detailed clinical data are shown in Table 1.

**Table 1:** Clinical data of all patients.

Patient number	sex	Age (y)	mRS (p)	NIHSS (p)	Barthel (p)	Stroke	TIA	GoS (%)	GoS (%)	GoS(%)	GoS(%)
								ICA re.	ICA li.	MCA re.	MCA li.
1	f	66	0	0	100			100			
2	m	63	0	0	100			70–80			
3	f	70	0	0	100		x		80		
4	f	79	2	3	100		x		70–80		
5	m	45	0	0	100	x			100		
6	f	45	1	1	100	x					100
7	f	56	0	0	100	x			100		
8	m	42	1	1	100		x			100	
9	f	50	0	0	100			100			
10	m	59	1	2	100	x		90			
11	f	57	0	0	100	x			100		
12	f	45	0	0	100		x	70			
13	m	63	2	4	100	x		80			
14	f	49	0	0	100	x		90			
15	f	36	2	2	100	x			100		
16	m	76	0	0	100	x		70			
17	m	31	0	0	100	x			100		
18	m	47	2	4	75	x		100			
19	f	63	0	0	100	x				70	
20	f	37	0	0	100				100		
21	f	73	0	0	100			70	30		
22	m	48	0	0	100				100		
23	m	46	0	0	100	x			100		
24	f	73	0	0	100		x		70–80		
25	m	65	0	0	100				100		
26	f	74	0	0	100			80			
27	m	58	0	0	100		x		>80		
28	m	68	0	0	100	x		70–80			

y: years; p: points; mRS: Modified Rankin Scale, NIHSS: National Institute of Health Stroke Scale; GoS: Grade of Stenosis; ICA: Internal Carotid Artery; MCA: Middle Cerebral Artery.

In the visual qualitative analysis, 11/28 ASL images were rated uninterpretable owing to severe patient motion artifacts, no ASL series was rated of good quality. In DSC-MRI in contrast, 13/28 image series were rated of good quality and only 1/28 DSC-TTP maps was rated as uninterpretable. In detail (good/medium/sufficient/uninterpretable): ASL (0/4/13/11); DSC-CBF (13/15/0/0), DSC-TTP (13/12/2/1). For the following analysis, patients with ASL measurements rated as “uninterpretable” were excluded resulting in 17 patients. 14/17 patients showed delay in DSC-TTP ipsilateral to the stenosis. In 10 of these 14 patients, ASL also showed delay by an arterial transit delay artifact (ATDA) (sensitivity: 71%). In DSC-CBF, 11/17 patients showed ipsilateral hypoperfusion. Corresponding ASL images identified ATDA in 9 of these patients (sensitivity: 81%). In ASL, hypoperfusion always occurred as part of ATDA, no isolated hypoperfusion without ATDA was found in ASL. Detailed results of the qualitative assessment are found in Table 2 and Table 3.

**Table 2:** Visual rating of DSC-CBF/ASL.

		DSC-CBF		
		hypoperfusion	normal	
ASL	ATDA	9	3	(12)
	normal	2	3	(5)
		(11)	(6)	

Results of the visual qualitative analysis. As hypoperfusion in ASL occurred exclusively as part of ATDA and not isolated, for ASL only the categories ATDA and normal are presented.

Rows show imaging findings for ASL-CBF, columns show imaging findings for DSC-CBF. Patients rated uninterpretable were excluded.

Of 11 patients with hypoperfusion in DSC-CBF, 9 patients showed ATDAs in ASL (specificity: 82%; false negative rate: 18%). 6 patients had normal DSC-CBF findings, 3 of them showed ATDAs in ASL (false positive: 50%).

DSC-CBF: Dynamic Susceptibility Contrast - Cerebral Blood Flow; ASL: Arterial Spin Labeling; ATDA: Arterial Transit Delay Artifact.

**Table 3:** Visual rating of TTP/ASL.

		TTP		
		delay	normal	
ASL	ATDA	10	2	(12)
	normal	4	1	(5)
		(14)	(3)	

Results of the visual qualitative analysis. As hypoperfusion in ASL occurred exclusively as part of ATDA and not isolated, for ASL only the categories ATDA and normal are presented.

Rows show imaging findings for ASL-CBF, columns show imaging findings for DSC-TTP. Patients rated uninterpretable were excluded. Of 14 patients with a TTP delay, 10 showed Arterial Transit Delay Artifacts in ASL (specificity: 71%; false negative rate: 29%). Out of 3 patients with normal findings in TTP, 1 showed ATDAs in ASL (false positive rate: 67%).

DSC-TTP: Dynamic Susceptibility Contrast - Time to Peak; ASL: Arterial Spin Labeling; ATDA: Arterial Transit Delay Artifact.

**Figure 3:**
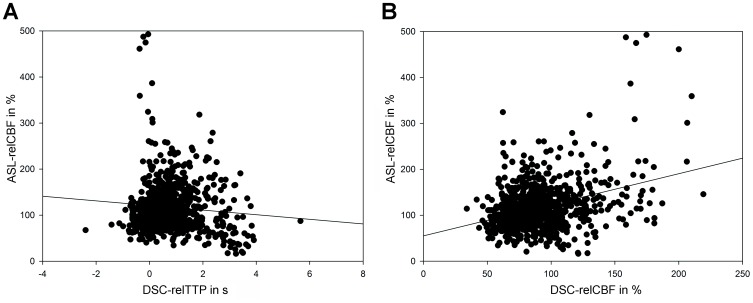
Scatter plots comparing DSC-relCBF and ASL-relCBF (A) and relTTP and ASL-relCBF (B). Pooled data of 17 patients. The diversity of the results is demonstrated in the two scatter plots. Spearman‘s rho for the pooled data was r = 0,24 (p<0,05) for DSC-relCBF vs ASL-relCBF and r = −0,32 (p<0,05) for relTTP vs ASL-relCBF.

**Figure 4:**
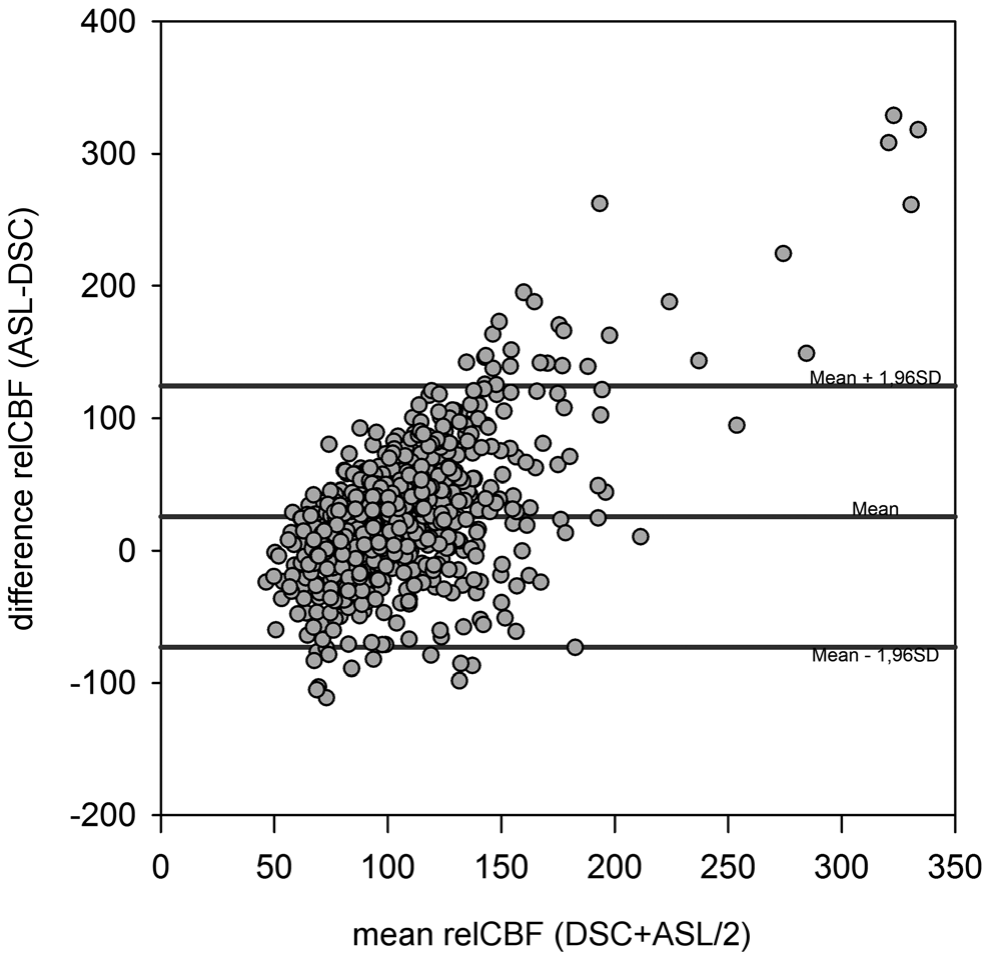
Bland-Altman-Plots comparing DSC-CBF and ASL-CBF. In the Bland Altman Plot, there is a large positive difference around 144% for high mean relCBF values between the two modalities. For low mean relCBF values, on the other hand, a large negative difference around −113% is seen. This pattern is suggestive of a proportional error. However, this was not confirmed by a logarithmic Bland-Altman Plot (not shown).

In the quantitative analysis, pooled data showed a weak correlation for DSC-relCBF vs ASL-relCBF (r = 0.24 [p<0.05]) and a weak negative correlation for DSC-relTTP vs ASL-relCBF (r = −32 [p<0.05]). Scatter plots are shown in Figure 3. The Bland Altman Plot showed a mean difference between DSC-relCBF and ASL-relCBF measurements of 13% relative difference with a high spread of values ranging from –113% to +144% relative difference. Negative differences were seen in low average relCBF and high differences in high average relCBF, suggesting a proportional error. This was, however, not confirmed in a logarithmic transformation of the plot (data not shown). The Bland Altman Plot is shown in Figure 4.

TTP-ROIs with a corresponding ATDA on ASL had a significantly (p<0.001) longer median delay (1.9 sec) than those without a corresponding ATDA (0.6 sec).

In the volumetric grey matter analysis, for ASL-CBF and DSC-CBF no significant correlation was found for all three arterial flow territories (ACA: r = 0.01 [p = 0.97], MCA: r = −0.09 [p = 0.72], PCA: r = −0.05 [p = 0.85]).

## Discussion

We report on the performance of an arterial spin labeling product sequence to measure CBF in patients with steno-occlusive disease in comparison with the clinical standard DSC-MRI. The tested ASL sequence is the only commercial, approved product sequence currently available for this system [10]. Sensitivity to patient motion made 39% of obtained ASL-images uninterpretable. In the visual analysis, ASL identified blood flow delay as defined by DSC-MRI with fair sensitivity, but led to unreliable measurement of CBF.

There is emerging consensus that ASL is a reliable alternative to exogenous contrast agent based perfusion measurements. Its use in routine clinical imaging has been encouraged [16]. However, most of the ASL techniques used for scientific neuroimaging are research sequences. This is important to note, as these sequences are usually not available in the clinical setting. Clinicians, who implement ASL in routine clinical imaging, currently are obliged to use commercially available product sequences. Therefore, the need for a validation of those sequences for CBF measurements in specific neurovascular pathologies is demanded [9], [10]. For cerebrovascular disease, only a few clinical validation studies have been conducted with product sequences[17]–[22]. Particularly for steno-occlusive disease, validation studies have not been performed for all available product sequences. Further, ASL in steno-occlusive disease is of high interest as it can be used in patients with contraindications for gadolinium and facilitates a noninvasive estimation of the cerebrovascular reactivity (CVR) [23]. In this respect, we aimed to define the performance of a commercially available ASL sequence (PICORE, pulsed ASL [PASL] technique) in patients with unilateral hemodynamically relevant steno-occlusive disease on a widely available 3T MRI system.

**Figure 5:**
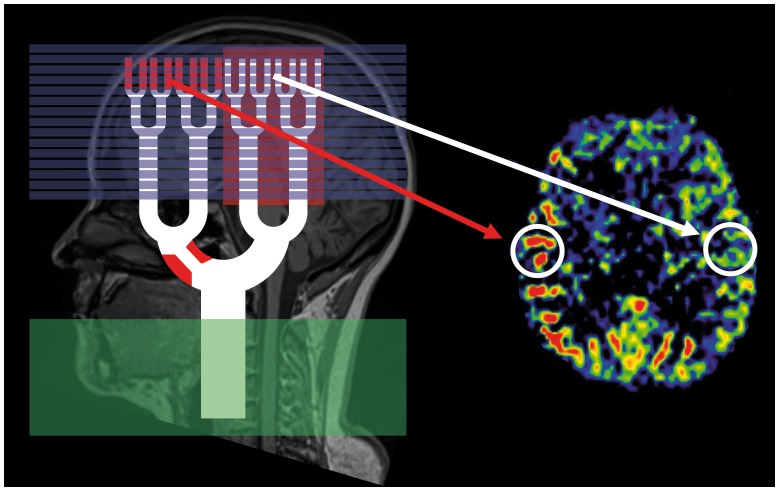
Arterial Transit Delay Artifact. Origin of the Arterial Transit Delay Artifact in ASL (labeling slice = green, imaging slices = blue). If arterial transit time is prolonged as it is the case in our patients, the inflow time is too short. Imaging slices capture high signal of labeled blood that is still in small arteries (as indicated by hyperintensities inside the labeling slices; red arrow) and hypointense areas, which the labeled blood has not yet reached. In parts of the brain where the arterial transit time is normal, blood has already exchanged with residual water and the ASL image appears normal (white arrow).

**Figure 6:**
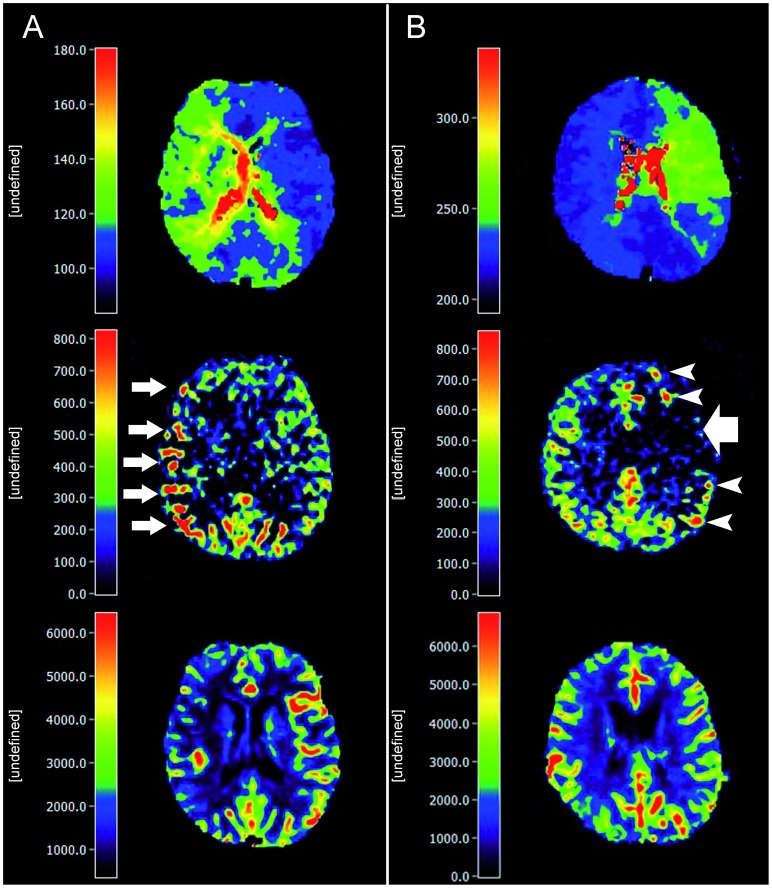
Exemplary patients showing blood arrival delay effects in ASL imaging. A) 66-year-old female, occlusion of the right ICA. The TTP image shows a visible ipsilateral delay (relTTP = 0.8 sec). In the ASL image, hyperintense arterial transit delay artifacts are seen (small white arrows). On the reference DSC-CBF map, a hypoperfusion in the affected hemisphere is present. B) 45-year-old female, occlusion of the left middle cerebral artery. In the ASL image, a hypointensity is seen in the area affected by delay (large white arrow, relTTP = 1.5 sec). In contrast, no apparent changes are present in the DSC-CBF map. In patient A, a moderate blood transit delay leads to the presence of hyperintense ADTA. The more severe delay in patient B might explain the hypointense ADTA surrounded by hyperintense areas in its borderzone as a sign of collateral macrovessels. All slices are coregistered. The scales do not represent absolute values.

In PASL, the spins of protons of passing blood are inverted by a radiofrequency (RF)-pulse at a labeling slice usually at the neck, covering the internal and external carotid and the vertebral arteries. By this technique, a single bolus of labeled blood is generated. The time this blood bolus needs to reach the brain capillaries is termed arterial transit time (ATT). In the brain capillary bed, labeled blood water then exchanges with residual tissue water. ASL images are acquired at one predefined time point after labeling, the inflow time (TI). It is important to notice that the TI has to be longer than the ATT to allow imaging in the capillary phase of blood transit. A second (control) image is then obtained without labeling. Subtraction of the two images yields perfusion weighted maps providing CBF-values. All ASL sequences in steno-occlusive disease, however, faces major obstacles owing to the prolonged ATT caused by stenosis/occlusion of brain feeding arteries. In this case, two signs may be observed: *First*, at the predefined inflow time, the blood bolus has not yet reached the capillary bed in the regions affected by steno-occlusion. Therefore, labeled blood is found mainly in precapillary arterioles. On ASL images, these vessels appear as hyperintense dots or lines depending on the image orientation [10], [13], [24]. *Second*, if the delay leads to very long arterial transit times, the blood bolus has not yet reached the perfusion territory at the predefined inflow time at all. In these regions, the signal is very low or lacks completely and thus severly underestimates real perfusion [10], [25]. These signs represent the arterial transit delay artifact (ATDA) [10], [13], [24]. The origin of the ATDA is shown in figure 5, examples for both effects are shown in figure 6. Both effects may appear simultaneously when areas with different prolonged ATTs are present in one patient. Therefore, a patient with normal cerebral blood flow but delayed arrival time, might have a falsely elevated or a falsely decreased CBF in the ASL image (Figure 6). In line with pathophysiology, these effects were present in our study since ipsilateral ATDA was found in 12 of 17 patients (71%). On one hand, the presence of ATDAs clearly identified the site of DSC-TTP-delay and/or DSC-hypoperfusion in the qualitative analysis and areas with ATDA in ASL showed a longer median TTP-delay on TTP-maps. On the other hand, however, the hyperintense nature of ATDA led to an unreliable quantification of ASL-CBF. This causes a weak correlation between ASL-CBF and DSC CBF-values as indicated by BA-plot analysis. These results at 3 T are supported by previous results obtained at 1.5 T, Wolf et al. found no correlation between ASL-CBF and DSC-CBF at 1.5 T in patients with steno-occlusive disease [13]. In our ROI-based approach we chose large ROIs to account for spatial distortion of the different imaging modalities. As a consequence, our ROIs included white matter, in which ASL might not allow for reliable quantification of CBF. However, our volumetric analysis of gray matter perfusion alone did not lead to an improved correlation.

To account for the prolonged ATT in patients with steno-occlusive disease it would be possible to choose longer TIs [26], but most of the signal would be lost at that time [27]. This intrinsic problem can only be solved by multiple averaging but leads to unacceptable scanning times.

Out of 28, 11 data sets (39%) were uninterpretable owing to patient motion artifacts despite online motion correction (performed by 3D-PACE) and, generally, image quality in ASL was worse in comparison with DSC-MRI. Patients were asked to refrain from motion, and small pads were placed between the patient head and the coil to limit head movement. The high frequency of motion artifacts in our study - despite the mentioned measures, - might thus reflect a limitation of this method in our specific patient sample, as increased patient motion reflects clinical reality. This is supported by a validation of the same product sequence in acute stroke, where the authors also identified patient motion and imperfect motion correction as a major factor for limited image quality [18]. Offline motion correction by in-house solutions might be preferable [28] but is not available in the clinical setting.

The described PICORE sequence offers the option of crusher gradients, that suppress intravascular blood signal [29] and reduce hyperintense vessel ATDAs. However, for reasons of methodology we did not choose this option: *First*, it does not eliminate the cause of the artifact, but its visibility. *Second*, the ATDA indicates the underlying transit delay and the lack of this information would render image analysis more difficult. *Third*, the use of crusher gradients further reduces the signal-to-noise ratio in ASL, which is already intrinsically low [10] especially in sequences without background suppression, e.g. the PICORE sequence.

Therefore, the PICORE product sequence available for the MRI system used in our study is not suited to reliably depict CBF in steno-occlusive disease at 3 T owing to the sensitivity to delayed blood arrival.

Currently, new ASL sequences address many methodological issues by correction for the delayed blood arrival time employing measurements at multiple inflow times. A very promising approach is a 3D ASL sequence (3D-GRASE) [30]. The benefit of this sequence in patients with steno-occlusive disease has been shown recently [31]. Another promising approach is the “look-locker approach” (ITS-FAIR [32], QUASAR [33]). Using this sequence in steno-occlusive disease at 3 T, recent studies found a high correlation with SPECT data [17] and a fair [34] to high [35] correlation with H2O-PET. These sequences, however, are not yet commercially available for all MRI systems.

Our study has some limitations: First, perfusion changes cannot be ruled out completely given the heterogeneity of perfusion alterations in patients with steno-occlusive disease. However, DSC-imaging followed ASL immediately and the clinical status of the measured patients was stable. Second, the methods of ASL and DSC differ considerably: While ASL uses blood as a freely diffusible and endogenous tracer, gadolinium in DSC mainly remains intravascular. Such methodological differences are a well-known difficulty of multimodal imaging and must be kept in mind when interpreting the results of comparative studies. While DSC-MRI can be considered a clinical standard for perfusion imaging, it is not a gold-standard. Methodological shortcomings include overemphasis of large vessels intrinsic to GRE-imaging [36] and the influence of the deconvolution method in patients with transit delay [37]. While a block circulant deconvolution (cSVD) might be preferable to sSVD from a technical point of view, this has been shown in direct comparison in only few pilot studies [37], [38]. Additionally, cSVD requires much more computing power limiting its use in the clinical setting. Therefore, sSVD, as the current clinical standard, was chosen for the comparison with a clinically available ASL sequence in this study.

In conclusion, the commercially available ASL-sequence used in our study can indicate delay of blood arrival in steno-occlusive disease, but cannot be recommended for CBF measurement in these patients. New ASL-sequences present promising alternatives, but are not yet available for clinical use on all MRI systems.
